# Peer teachers as ultrasound instructors? – a systematic literature review of peer teaching concepts in undergraduate ultrasound education

**DOI:** 10.1186/s12909-024-06345-7

**Published:** 2024-11-26

**Authors:** Lone Winter, Ricarda Neubauer, Johannes Weimer, Christoph F. Dietrich, Agnes Wittek, Lina Schiestl, Milka Marinova, Valentin Sebastian Schäfer, Brigitte Strizek, Florian Recker

**Affiliations:** 1 Department of Obstetrics and Prenatal Medicine, University Hospital Bonn, Venusberg Campus 1, Bonn, 53127 Germany; 2 Rudolf Frey Learning Clinic, University Medical Centre of the Johannes Gutenberg University Mainz, Mainz, Germany; 3 Department General Internal Medicine (DAIM), Hospitals Hirslanden Bern Beau Site, Salem and Permanence, Bern, Switzerland; 4 Department of Gynecology and Obstetrics, University Hospital, Johannes Gutenberg University Mainz, Mainz, Germany; 5 Department of Nuclear Medicine, University Hospital Bonn, Venusberg Campus 1, Bonn, 53127 Germany; 6 Department of Rheumatology and Clinical Immunology, Clinic of Internal Medicine III, University Hospital Bonn, Bonn, Germany

**Keywords:** Student ultrasound education, Peer teaching, Peer-assisted learning, Train the teacher, Peer teacher training concepts

## Abstract

**Background:**

As ultrasound is one of the most utilized imaging procedures in clinical practice in Germany, its integration into undergraduate medical education is imperative. Thereby, the limited availability of qualified instructors is a major challenge. Peer tutors, who are trained to instruct their peers collaboratively, could resolve staff constraints. This systematic review explores the literature on peer teaching in undergraduate ultrasound education, aiming to provide an overview of methodologies, outcomes, and peer teacher training concepts.

**Methods:**

Following the PRISMA guidelines, a systematic literature review was conducted on the subject of peer teaching in undergraduate ultrasound education. Using PubMed and Google Scholar as databases, studies in English or German involving training concepts for peer teachers in undergraduate ultrasound education, published up to November 21, 2023, were included. Data extraction of original studies followed the PICOS schema with special respect to didactic concepts of peer tutor training programs and the effectiveness of peer teachers compared to faculty instructors. A modified version of the Newcastle–Ottawa Scale (NOS) was used to assess the quality of included studies.

**Results:**

Finally, the search resulted in 20 relevant original studies, including 16 studies exploring peer teacher training concepts. Predominantly, peer teachers studied in their 4th year of medical school and on average one year further compared to their students. Peer teacher training was integrated into curricula by course-based concepts (93.8%) and internships (50.0%). Didactic modalities varied, encompassing laboratory rotations including the scanning of patients, the scanning of fellow students, lectures, and didactic training. The median training duration was about ten days. Of six comparative studies, five found peer-assisted learning to be comparably effective and one even better than faculty-led courses.

**Conclusion:**

Despite the growing amount of literature underlining the effectiveness and wide application of peer teaching in ultrasound education, training concepts stay heterogenous without a standardized system for training and qualifying peer teachers. Developing comprehensive guidelines for peer tutor education could increase acceptance and recognition of peer-assisted learning and ensure minimum training standards.

## Introduction

Given its numerous advantages, ultrasound plays a crucial role as a diagnostic tool in everyday clinical practice and became the most frequently used imaging procedure for extended clinical examination in Germany [1]. As a radiation-free, non-invasive, and cost-effective modality, it also meets the ideal conditions for medical students to practice a commonly used clinical imaging tool early in their studies [2, 3]. For preclinical students, ultrasound training facilitates the understanding of anatomy and physiology, and strengthens the acquisition of diagnostic and procedural skills by the focused extension of physical examination [4–6]. Furthermore, early exposure to ultrasound during medical school provides the chance to decrease the educational load during residency [7]. Both the European Federation of Societies for Ultrasound in Medicine and Biology (EFSUMB) and the World Federation of Ultrasound in Medicine and Biology (WFUMB) provide recommendations for the vertical integration of ultrasound training into preclinical and clinical medical education in terms of a longitudinal curriculum [8–10]. The EFSUMB suggests the implementation of preclinical courses to improve students’ understanding of anatomy, physiology and pathology, and clinical courses to strengthen students’ diagnostic ultrasound competencies [8]. In addition, the WFUMB recommends at least 40 h of practical ultrasound training during preclinical medical education [9]. However, findings by Nourkami-Tutdibi et al. reveal that students perceive current ultrasound education as insufficient and report limited ultrasound-related knowledge and skills among medical students [7]. An underlying problem might be potential cognitive overload by the complexity of ultrasound examinations, requiring theoretical knowledge about the physics of ultrasound and anatomy, image interpretation, pattern recognition for pathologies, communication skills, and the ability to use the device correctly to obtain high-quality images [11]. While theoretical content can be efficiently taught in lectures or using scripts, the actual scanning requires hands-on practice under qualified instructor supervision in a low student-instructor ratio [10]. The shortage of qualified instructors, the lack of teaching time slots, and the costs of sonography equipment, continues to limit the further curricular incorporation [12–14]. However, the use of peer-assisted learning offers a chance to resolve these barriers [3, 15–17]. “Peer teaching” or “peer-assisted learning” comprise various approaches of instruction between students who are at a different (´near-peer teaching´) or the same academic level (´peer-to-peer-teaching´) [18]. For this review, peer teaching is defined as the non-hierarchical, collaborative instruction of students to fellow students to acquire knowledge and skills by theoretic and practical ultrasound [19, 20]. There are few approaches described in the literature to train peer teachers. In this context, the following systematic review of the literature aims to provide an overview of published studies on the implementation of peer teaching in undergraduate medical ultrasound education. Furthermore, different concepts for training students to become peer teachers are investigated and experiences of courses led by peer teachers compared to faculty instructors are displayed.

## Methods

### Search strategy

**Fig. 1:**
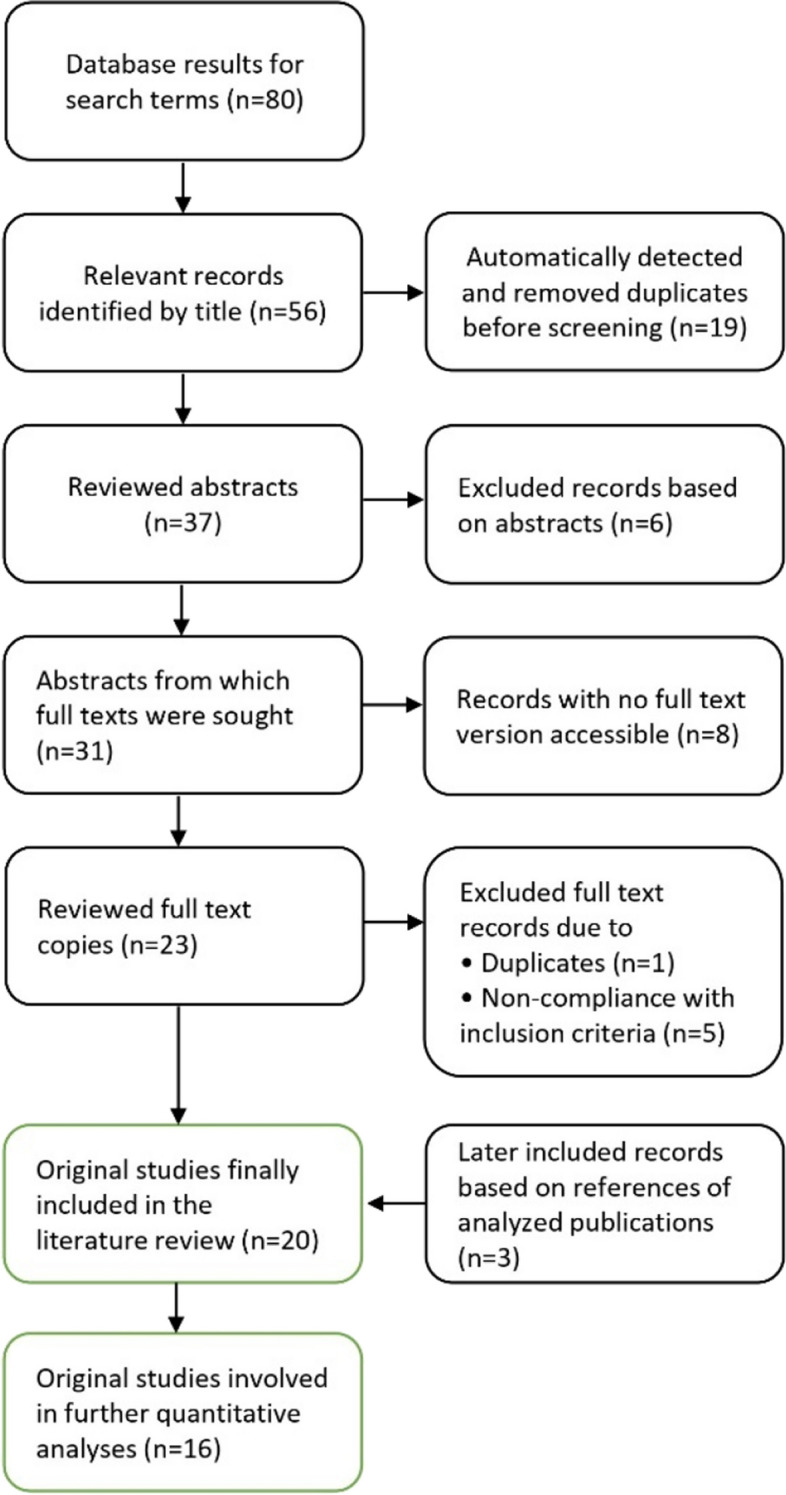
Literature selection process according to the Preferred Reporting Items for Systematic Reviews and Meta-Analyses (PRISMA) statement [21, 22]

This systematic search of the literature was conducted in accordance with the updated Preferred Reporting Items for Systematic Reviews and Meta-Analyses (PRISMA) statement on reporting systematic reviews and meta-analyses of studies [21, 22] (see Fig. 1). A literature search on the topic of peer teaching concepts in ultrasound education was conducted in PubMed and Google Scholar using the keywords [ultrasound] and [peer] combined with [undergraduate], [education], [teaching], or [curriculum]. All studies published in English or German up to November 21, 2023, that investigated ultrasound training with peer teachers, were eligible for inclusion. First, publications were selected according to the level of information based on their title. Duplicate entries were removed automatically. Subsequently, three authors (L.W., R.N. and F.R.) independently screened the publications’ abstracts for compliance with predetermined eligibility criteria, using a blinded approach. Any differences among the authors concerning inclusion were resolved through direct discussion. After the removal of excluded records based on abstracts, full-text versions were obtained, read, and analyzed for data extraction.

### Eligibility criteria

All relevant publications addressing the topic of peer teaching in undergraduate ultrasound education were included. Special interest was given to the didactic structure of peer teacher training formats. Both randomized controlled trials and observational studies were eligible, as were comparative studies that evaluated the effectiveness of peer-led versus faculty-led instruction. After screening studies for publication type, case reports and expert opinions were excluded due to their limited generalizability. Furthermore, relevant publications that were referenced by included studies were additionally involved. Studies were selected for their relevance to undergraduate medical education and the quality of the reported training interventions. This approach aims to provide a comprehensive overview of the current literature on the implementation of peer teaching in undergraduate allopathic medical education. It analyzes various training concepts for peer teachers and compares the experiences and effectiveness of peer teaching with faculty-led instruction. The literature search was limited to undergraduate ultrasound education due to differing didactic objectives regarding postgraduate settings. Furthermore, articles were excluded due to duplication, non-fulfillment of inclusion criteria, inaccessibility of full-text versions or published in languages other than German or English.

### Data extraction and synthesis

All included reports were analyzed for the targeted study characteristics, according to pre-defined characteristics categorized after the PICOS (Participants, Intervention, Comparator, Outcome, Study design) schema (see Table 1) [22]. For this purpose, medical students were defined as participants. To ascertain the students' theoretical and practical competence profile during the ultrasound course, their academic year and assignment to the preclinical or clinical study period were analyzed. The students participated in differently designed ultrasound courses led by peer teachers. The learning outcomes of students instructed by peer teachers were compared with those of students who had participated in courses with varying structures, instructors, or didactic approaches. To offer an overview of the diverse didactic concepts employed in the training of peer teachers, the educational approaches in terms of timing (year of medical school and study period), format, content, duration, as well as the involvement of supervisors and post-training exams were investigated. Furthermore, comparative studies on the learning success achieved by peer or professional instruction were analyzed. Two of the authors read and extracted data independently. No automation tools were used. For data synthesis, relevant data was collected and compared in tables. All descriptive statistical analyses were performed in Microsoft Excel for Windows (Version 2311 Microsoft Corporation, Redmond, Washington, USA) and IBM SPSS 29.0 for Windows.

**Table 1:** Study characteristics for included studies according to PICOS

**P**articipants	Students pursuing medical degrees
**I**ntervention	Peer-teaching in ultrasound education
**C**omparator	Different course concepts, instructors, and educational approaches
**O**utcomes	Assessment and evaluation of peer teacher training programs, peer teachers’ competency, students’ learning outcomes, further didactical parameters, and further key messages
**S**tudy design	Original studies or reports on prospective studies, interventional studies, observational studies, and cross-sectional studies

### Quality assessment of included studies

The quality assessment of included studies was based on a modified version of the Newcastle–Ottawa Scale (NOS) [23]. This scale is traditionally used for evaluating the quality of non-randomized studies, particularly clinical cohort studies, and is recognized for its rigorous approach [24]. The included didactic teaching studies exhibited non-standardized, heterogeneous study designs with characteristics not comparable and not transferable to clinical cohort studies. We therefore adapted the original NOS of clinical cohort studies to the requirements of the included prospective interventional and observational teaching studies investigating peer-teacher-led ultrasound education in undergraduate medical education.

The scale consists of three categories to evaluate the methods of included studies as well as to assess the findings' relevance and significance: 1) selection (maximum of four scores), 2) comparability (maximum of two scores), and 3) outcome (maximum of three scores) (see Appendix Table 7). A total score of seven or higher indicates good quality of the study. Scores between five and six suggest fair quality, while scores below five suggest poor quality of the study [25]. The scoring algorithm of each category is shown in Appendix Table 8.

The modifications made to the NOS include the following aspects.

1. Selection Criteria: The items related to the selection of study groups assess the representativeness of the included cohorts. The exposed cohort was defined as medical students taught by peer teachers, whereas the non-exposed cohort was defined as those taught by faculty. As for the ascertainment of exposure, we included criteria to evaluate the applied peer teacher training concepts. This modification aimed to ensure peer teachers' content-related, practical, and didactic competence, as well as to verify the quality of training provided by peer teachers. Furthermore, by demonstrating the absence of prior ultrasound skills, it is ensured that the outcome of interest was not present at the start of the study.
2. Comparability Factors: We modified the assessment of comparability to include whether the study controls for factors such as the year of medical school, age, and sex of study participants, or if a randomized cohort allocation is conducted.
3. Outcome Evaluation: We refined the outcome assessment criteria to evaluate the reliability and objectivity of the methods used to assess acquired ultrasound skills, as well as the appropriateness of follow-up examinations for assessing long-term outcomes.

By detailing these modifications and their rationale, we aim to enhance the reliability of our quality assessment and improve the replicability of our findings.

## Results

### Search results

The literature search yielded 80 records, with 56 deemed relevant based on the title. In total, 39 records were excluded due to duplication, inappropriate abstract content, inaccessibility of full-text version, and non-compliance with inclusion criteria after full-text review. After additionally including three further records based on references of analyzed publications, the overall search produced a total of 20 publications dedicated to peer teaching in medical undergraduate ultrasound (see Fig. 1). These 20 literature entries represent original studies encompassing a diverse range of peer teacher-led course concepts and were examined according to the PICOS scheme with regard to the participants' expertise levels and various aspects of the curricular format in ultrasound training programs (see Table 2). Furthermore, in 16 of the 20 original studies included, the didactic approaches employed to train peer teachers were described, as outlined in Table 3.

**Table 2:** Original studies analyzed according to the PICOS schema

**Authors, Country of origin**	**Title and year of publication**		**Participants**				**Intervention**	**Comparator/ Control group**	**Outcome**	**Study design**
			**Number (n), Year of medical school (y°) of total study years (y**^t^**), preclinical (pc) or clinical (c)**						**Study outcome and key message on peer teaching**	
			**n**	**y°**	**y**^t^	**pc/c**				
**Ahn et al. **[26], Colorado	Training peer instructors for a combined ultrasound/ physical exam curriculum	2014	310	1^st^ - 2^nd^	4	pc	Peer-teacher led US and physical examination training	Faculty instructors (emergency medicine, internal medicine, family medicine, anesthesia, critical care medicine) leading a combined US/ physical examination course	For most of the sessions, peer teaching was perceived as effective as instruction by faculty. Senior students who have completed elective US training may serve as valuable resources if the access to skilled instructors is limited. Students participating in a 4-week elective demonstrate higher scores than those in a 2-week elective, suggesting a positive correlation between the duration of training and skill acquisition. The skill improvement in PT is considered as an asset for residency and clinical practice.	PS
**Ben-Sasson et al. **[16], Israel	Peer-teaching cardiac ultrasound among medical students: A real option	2019	66	n. sp.	7	c	Peer-teacher led cardiac US training	US training by faculty instructors (cardiologists, diagnostic medical sonographers)	Students who were taught by peer teachers scored better on most parts of the final examination. Peer teaching could help medical schools teach US techniques with minimal reliance on highly qualified faculty trainers.FI may suffer from the cognitive bias "curse of knowledge", which claims that experienced individuals are unable to share their knowledge with a novice listener, due to discrepancies between their perceptions. PTs proximity to their student peers in terms of experience and understanding of TTE, enables a focused approach to teach and a quick resolution of difficulties.	PS
**Boivin et al. **[6], USA	Evaluation of a Required Vertical Point-of-Care Ultrasound Curriculum for Undergraduate Medical Students	2022	439	1^st^ - 4^th^	4	pc + c	Implementation of a vertical POCUS curriculum using handheld US devices, peer teaching and a flipped classroom approach including virtual learning modules, gamification and station rotations	-	Handheld US devices, near-peer teaching, flipped classroom formats, gamification, and staggered small group scheduling enables hands-on practice and low student-to-instructor ratios. The combination of peer teaching, low student-to-faculty ratio, and handheld US in US curriculum allowed for an improvement in students’ US knowledge and skills.	PS
**Celebi et al. **[27], Germany	Outcomes of three different ways to train medical students as ultrasound tutors	2019	44	3^rd^ - 5^th^	6	c	Training of peer teachers by a combination of an US course and internship	a) Training of peer teachers by an US course only b) Training of peer teachers by an internship only	Both training programs of the peer teachers achieved comparable learning outcomes to train medical students as peer tutors. All PT no matter their training approach improved their theoretical knowledge and their practical scanning skills significantly and to a comparable degree.	PS
**Celebi et al. **[3], Germany	Development and implementation of a comprehensive ultrasound curriculum for undergraduate medical students - a feasibility study	2019	160	5^th^	6	c	Peer-assisted hands-on sessions during US curriculum	Faculty members	Using a hybrid concept, following national guidelines, the students are trained with a course to ensure that all the critical content is covered, added to a four-week rotation through seven different US laboratories so that the students encounter considerable scanning practice and can experience some of the pathologies in real life. The developed course was feasible and well accepted by the volunteer students with the student tutors rated as good as the faculty members.	PS
**Celebi et al.**[28], **Germany**	Three different ways of training ultrasound student-tutors yield significant gains in tutee's scanning-skills	2019	75	5^th^	6	c	Ultrasound course for medical students taught peer teachers who were trained either by a course-based approach, an internship or a combination of both	Outcomes of peer teaching with prior a) Course-based training b) Internship training c) Course + Internship	All peer teacher training approaches led to comparable learning success among the participants. This indicates that all methods are suitable for training students to become peer teachers. When selecting the training format, the individual advantages and disadvantages of each concept should be weighed up against the available resources.	PS
**Dickerson et al. **[29],UK	The role for peer-assisted ultrasound teaching in medical school	2017	105	3^rd^	6	c	Peer-teacher led FAST training	-	Medical students are willing to be taught by fellow students. They report a knowledge gain in US and therefore recommend the course.	PS
**Eimer et al. **[19],Germany	Video-based, student tutor- versus faculty staff-led ultrasound course for medical students - a prospective randomized study	2020	96	4^th^	6	c	Peer -teacher led US course using a video-based approach	Conventional, faculty-led US course without videos	Students of both study cohorts showed comparable learning effects indicating that peer-teacher led ultrasound course with video-based media supply can offer an effective solution for staff restraints. Nevertheless, motivation of alle students for the videos alone was low, so either peer teachers or faculty instructors may still need to be present.	PS
**Hari et al. **[15], Switzerland	Describing Ultrasound Skills Teaching by Near-Peer and Faculty Tutors Using Cognitive Apprenticeship	2022	64	3^rd^	6	c	Peer-teacher-led 55 minutes hands-on US lesson as part of an US elective	Faculty instructor led control	US train-the-tutor programs should particularly focus on coaching and articulation. Near-peers’ similar use of teaching methods supports the use of near-peer teaching in US skills education.	PS
**Knobe et al. **[30], **Germany**	Undergraduate Curricular Training in Musculoskeletal Ultrasound: The Impact of Preexisting Anatomic Knowledge	2010	87	1^st^, 4^th^	6	pc + c	Peer-teacher and faculty led course with preclinical students without prior anatomy knowledge	Peer-teacher and faculty led course with clinical students with prior anatomy knowledge	While there was no significant difference between the faculty-led courses, the preclinical students without prior anatomical knowledge who were trained by students performed significantly worse. Professional instructors may therefore be more suitable for preclinical courses with students without prior anatomical knowledge.	PS
**Kühl et al. **[31],Germany	Student tutors for hands-on training in focused emergency echocardiography – a randomized controlled trial	2012	30	4^th^-5^th^	6	c	Focused emergency echocardiography course led by peer teachers	Focused emergency echocardiography course led by faculty instructors	Both groups showed an increase in learning, but there was a significant difference between the course groups. Peer teaching in echocardiography was inferior to instructor guidance. This may potentially be due to the complexity of echocardiographic examinations.	PS
**Li et al. **[32], USA	Comparing the Effectiveness and Image Quality of Musculoskeletal Ultrasound of First-Year Medical Students After Training by Student Tutors Versus Ultrasound Instructors: A Pilot Study	2022	24	1^st^	4	pc	Peer-teacher-led musculoskeletal US workshop	Musculoskeletal US workshop led by faculty instructors or senior medical students with more than five years of US experience	There was no significant difference in confidence and identification accuracy between the peer teacher and faculty instructor taught groups. However, students who were taught by faculty instructors performed better in the basic theory, which could indicate that theoretical parts of an ultrasound course should better be taught by professional lecturers.	PS
**Miller et al. **[5], USA	Near Peer POCUS Education Evaluation	2022	63	n. sp.	4	n. sp.	PoCUS instruction or simulation courses led by peer teachers	-	Peer teaching is the preferred learning method among students at this institution. Both peer teachers and students prefer peer teaching over faculty-led sessions. Both report an improvement in their US skills as a result of giving or participating in US courses.	PS
**Nourkami-Tutdibi et al. **[7], Germany	Long-Term Knowledge Retention after Peer-Assisted Abdominal Ultrasound Teaching: Is PAL a Successful Model for Achieving Knowledge Retention?	2020	40	3^rd^ to 5^th^	6	c	Investigating long-term effects of peer-assisted learning in US training	-	The peer-assisted approach resulted in retained knowledge and skills after a one-year-interval. It is an effective method of achieving a sustainable improvement in practical abdominal US skills and knowledge. Peer teaching can assure long-term knowledge retention.	PS
**Nourkami-Tutdibi et al. **[20], Germany	TEACHING MUST GO ON: flexibility and advantages of peer assisted learning during the COVID-19 pandemic for undergraduate medical ultrasound education - perspective from the "sonoBYstudents" ultrasound group	2021	75	n. sp.	6	n. sp.	Using peer-assisted learning concepts in US training during pandemic	-	Peer teaching during the pandemic enabled the same learning successes to be achieved as in previous pre pandemic semesters. US is a non-omittable part of medical skill training with easily appliable hygienic precautions during teaching sessions. An existing PAL concept can be adjusted to changing pandemic teaching circumstances.	PS
**Rong et al. **[33], USA	Effectiveness of Near-Peer Versus Faculty Point-of-Care Ultrasound Instruction to Third-Year Medical Students	2022	73	3^rd^	4	c	Peer-teacher-led PoCUS training	Faculty instructor (emergency care and critical care departments) led PoCUS training	The use of peer teaching is an effective tactic to maintain the high quality of education while working within resource constraints. Peer teaching was as effective as faculty-led teaching at PoCUS training of 3rd year medical students. FI displayed a higher competency and ability to create a comfortable environment. PT improved the students’ confidence with PoCUS.	PS
**Weimer et al. **[17], Germany	Undergraduate ultrasound training: prospective comparison of two different peer assisted course models on national standards	2023	888	3^rd^	6	c	10-week peer-assisted learning sonography course (20 teaching units)	Two-day peer-assisted learning sonography compact course (20 teaching units)	Sonography-specific competency was significantly more obtained by a course model stretching over several weeks than a compact course. PT-supported US education models can build US competencies. All participants were satisfied with the PT didactic skills.	PS
**Weimer et al. **[2], Germany	Long-Term Effectiveness and Sustainability of Integrating Peer-Assisted Ultrasound Courses into Medical School-A Prospective Study	2023	302	6^th^	6	c	Students at the start of their practical year, who attended a 10-week or two-day peer-assisted learning sonography course (20 teaching units) during the clinical part of medical school	Students at the start of their practical year, who did not attend a sonography course during the clinical part of medical school	At the start of their practical year, the participants have better practical and theoretical US skills than a control group who attended no US courses during their studies. Early US training can lead to long-term improvements in US skills. Peer-assisted US courses can sustainably increase both theoretical and practical competency of medical students. Integrating peer-assisted US training programs into the (early) clinical part of medical studies can create sustainable skills acquisition in the long term.	PS
**Weimer et al. **[34], Germany	FoCUS cardiac ultrasound training for undergraduates based on current national guidelines: a prospective, controlled, single-center study on transferability	2023	217	3^rd^ to 5^th^	6	c	Students who attended a peer-teacher-led 1-day FoCUS training course (12 teaching units)	Students who did not attend a FoCUS training course	The participants experienced significant subjective and objective skill improvement. The students were satisfied with the course approach, teaching materials, and tutors. Positive feedback from the participants in relation to the tutors’ ability to communicate, their presentation, and their teaching skills shows that structured, qualitative training in FoCUS is feasible and indispensable.	PS
**Yan et al. **[35], **Canada**	Sonoist: An Innovative Peer Ultrasound Learning Initiative on Canadian Teaching Hospital Wards	2022	20	1^st^ to 4^th^	4	pc + c	Peer-teacher-led US sessions	-	Peer-to-peer PoCUS teaching improved medical students’ clinical knowledge as well as sonographic skills and knowledge. Learning US was useful for correlating skills with physical exam and clinical diagnosis. Learners stated their preference for PT over FI.	PS

*Abbreviations:* *US* Ultrasound, *PoCUS* Point of care ultrasound, *TTE* Transthoracic echocardiogram, *FoCUS* Focused cardiac ultrasound, *PS* Prospective study, *MCQ* Multiple choice questionnaire, *n. sp.* not specified

**Table 3:** Ultrasound peer teacher training concepts

**Study**	**Training format**	**Time **	**Pre-existing experiences and if required (if yes - **✗)		**Course structure**						**Type of applied US and detailed description of course content **	**Assessment after training**	**On-going training and further assessment of knowledge and skill retention**	**Evaluation and Comparison of Peer and Faculty Instructors**		
					**Theoretical training and teaching methods**		**Practical training on models or patients**		**Didactic training**	**Teachers**				**Evaluation method of fellow students trained by PT**	**PT vs FI**	**Further details**
**Ahn et al. **[26]	Course	2-4w	✗	2- or 4- week advanced US elective in emergency US	✗	Lectures E-Learning video review of scans Clinical rotation in emergency department	✗	P	✗ 3 h / w	Faculty	**POCUS ** **Mandatory course content: ** Introduction to US, sonographic organ identification, US in trauma, focused echocardiography, US-guided vascular access, aortic US, first-trimester pelvic US **Elective topics: ** US of soft tissue, musculoskeletal US, US-guided procedures, vascular compression US, US-directed resuscitation, ocular US	Feedback by supervisors	Test of knowledge retention after 6 months	Evaluation survey	» / < PT < FI in ocular US	Peer tutors who had undergone a longer preparatory elective in advance and who led more training sessions were rated as more effective tutors. An individual improvement in teaching was observed in students to taught at least 3 sessions.
**Ben-Sasson et al. **[16]	Intern-ship	16h	-		✗	Lectures Clinical rotation	✗	M & P	- / n. sp.	n.sp.	**Transthoracic echocardiography (TTE)** Introduction into US physics and principles of 2D imaging and Doppler effect, cardiac anatomy, views, left ventricle function assessment, normal and pathologic valve structures, pericardial effusion, inferior vena cava width variations. Hands-on scanning using portable US devices	- / n. sp.	-	Practical exam	PT > FI	18% of the participating students had prior experience in echocardiographic US.
**Boivin et al. **[6]	Course	2 w	✗	2-week emergency medicine-based ultrasound elective	✗	Videos Case presen-tation Clinical rotation	✗	M	- / n. sp.	Faculty	**PoCUS** in various clinical environments, sonoanatomy and sonopathology, anatomical planes and orientation, technique, clinical integration, and US-guided procedures	Knowledge test	-	Theoretical test, OSCE, Self-Assessment	-	3^rd^ and 4^th^ year students were used to teach younger classes, while further years were trained by faculty.
**Celebi et al. **[27]	Course	5d	-	33% previously been a tutor, 36% previous didactic training, 41% previous experience with US	✗	Lectures, Script	✗	M	✗	Faculty + PT	All received a script covering all topics and picture examples of pathologies: Physics, artefacts, handling of the US device, documentation, liver, gallbladder, bile ducts, retroperitoneal structures with vessels and lymph nodes, pancreas, spleen, peritoneal cavity, kidneys, bladder, uterus, systematic examination abdomen, throat, thyroid, jugular veins, carotid arteries, lymph nodes, basic color doppler, compression duplex sonography of the deep veins, thorax, lung, eFAST, FEEL	MCQ, OSCE	-	Theoretical test (MCQ), OSCE	-	-
	Intern-ship	21-35d			✗	Script	✗	P	✗							
	Course + Intern-ship	5d+21d			✗	Lectures, Script	✗	M & P	✗							
**Celebi et al. **[3]	Course + Intern-ship	36d	-	-	✗	Lectures	✗	M & P	✗	Faculty	Basic physics, handling of US device, image optimization, anatomy, scanning techniques and pathologies of liver, gallbladder, bile ducts, retroperitoneum, abdominal vessels, lymph nodes, pancreas, spleen, kidneys, bladder, uterus, and prostate, thyroid, jugular veins, carotid arteries, systematic examination of the abdomen, duplex sonography and compression eFAST, FEEL Didactic training: “role and responsibilities of a student tutor; creating an environment that supports learning; reflection and feedback; explaining and visualizing; activating teaching strategies; conveying practical skills; handling difficult teaching situations; and simulation of a teaching situation with video-feedback” [3]	MCQ, OSCE	n. sp.	Theoretical test (MCQ), OSCE, Evaluation survey	»	-
**Celebi et al.** [28]	Course, Intern-ship or combi-nation of both	5-21d			✗	Lectures Script	✗	M & P	✗	Faculty	Standardized 1,5 day-long didactic training Basic physics, handling of US device, image optimization, US anatomy and pathologies of liver, gallbladder and bile ducts, retroperitoneum, abdominal vessels, lymph nodes, pancreas, spleen, kidneys, bladder, uterus, prostate, FEEL, thyroid, jugular veins, carotid arteries, thoracic ultrasound, eFAST	n. sp.	n. sp.	Theoretical test (MCQ), OSCE, Evaluation survey	-	-
**Hari et al. **[15]	Course	6d	?	Near-peer teachers in the US had a median of one year of prior teaching experience and taught four hours per month, while faculty had an average of 4.5 years' experience teaching US and currently teach two hours per month.	?	n. sp.	?	n. sp.	✗	Faculty	**Abdominal US** Division in basic and advanced course, each training lasted 3 days	- / n. sp.	n. sp.	OSCE	» (analysis of videos of teaching)	Near-peer and faculty tutors used similar teaching strategies in ultrasound skills training, with near-peer tutors spending more time creating a safe learning environment and discussing knowledge gaps. Near-peer tutors were also less likely to correct students' probe positioning directly, instead often observed silently, possibly due to their awareness of limited content expertise.
**Knobe et al. **[30]	Course	1w	-	n.sp.	✗	Self-directed study of literature	-	-	-	**Self-directed**	**Musculoskeletal US** 30 min introductory session on the handling of the US device and subsequent self-directed review of literature	- / n. sp.	n. sp.	Theoretical test (MCQ), OSCE, Evaluation survey	FI » PT for students with anatomic knowledge FI > PT for students without	The peer teachers benefited most from the project, supporting the idea that teaching others reinforces learning.
**Miller et al. **[5]	Course	n. sp.	-	n. sp.	✗	E-Learning, Lectures	✗	n. sp.	- / n. sp.	**Faculty**	**POCUS,** **Course content n. sp. **	Feedback by supervisors, Evaluation, Self-assessment	n. sp.	Evaluation survey	-	-
**Nourkami-Tutdibi et al. **[7]	Course	2m	✗	Students needed at least two years of medical school remaining to serve as tutors long-term after training.	✗	Online lectures, E-Learning	✗	M & P	✗	**Faculty**	**Abdominal US, FAST** **• Online lectures and textbook (60h): ** basic knowledge of 2D and Doppler US; B-mode, M-mode, normal range of important parameters **• abdominal US** **workshop (30h):**drawings, videos, hands-on scanning on healthy individuals **• US elective (4 w):** hands-on scanning on clinic patients (100 abdominal US; 20 thyroid US) • **Didactic skill training (40h):**training and communication skills	OSCE, personalized feedback	n. sp.	Pre- /post-training and follow up OSCE, Evaluation survey	-	A skill retention test of 15 randomly selected participants of the peer-assisted ultrasound course showed similar skills compared to the post-OSCE.
**Nourkami-Tutdibi et al. **[20], Germany	Course	2m	-	n. sp.	✗	Online lectures, E-Learning	✗	M & P	✗	**n. sp. **	**Abdominal US, FAST, TTE** • **Online lectures and textbook (60h):** basic knowledge of 2D and Doppler US; B-mode, M-mode, normal range of important parameters • **abdominal US** **workshop (30h):**drawings, videos, hands-on scanning on healthy individuals • **US elective (4 w):** hands-on scanning on clinic patients (100 abdominal US; 20 thyroid US) • **Didactic skill training (40h):**training and communication skills	OSCE, personalized feedback	n. sp.	OSCE	-	Student tutors were highly motivated to teach ultrasound lessons, as it allowed them to provide valuable in-person instruction during the pandemic, enhance their responsibility, and actively contribute to curriculum development.
**Rong et al. **[33], USA	Course	2w	✗	2-week elective formal US training	?	n. sp.	?	n. sp.	-	n. sp.	**POCUS** Vertical mandatory ultrasound curriculum and additional ultrasound elective had to be completed by future peer teachers	n. sp.	n.sp.	Theoretical test (MCQ), OSCE, Evaluation survey	»	Students taught by NP-instructors had higher scores on both the theoretical tests, while those taught by faculty instructors scored higher on the OSCE.
**Weimer et al. **[17], Germany	Course, Internship	30h	-	n. sp.	✗	n. sp.	✗	n. sp.	✗	n. sp.	**Abdominal US, FAST** DEGUM-certified US-course, Performance of at least 100 examinations in an US laboratory	Practical assessment	n. sp.	OSCE, Evaluation survey	-	The trained peer tutors and participants rotated throughout the course sequences, allowing participants to interact with a variety of instructors. A 10-week peer-assisted sonography course led to significantly higher competence and subject knowledge scores compared to a two-day compact course, suggesting that extended course models may be more effective for sustained ultrasound training in medical students.
**Weimer et al. **[2], Germany	Course, Internship	30h	-	n. sp.	✗	n. sp.	✗	n. sp.	✗	n. sp.	**Abdominal US, FAST, Focused Cardiac Ultrasound (FOCUS)** • DEGUM-certified US-course • Performance of at least 100 examinations in an US laboratory • DEGUM-certified US-course • Performance of at least 50 examinations in an US laboratory	Practical assessment	n. sp.	Theoretical test, Practical assessment, Evaluation survey	-	-
**Weimer et al. **[34], Germany													n. sp.	Theoretical assessment, Evaluation survey		-
**Yan et al. **[35], Canada	Course, Internship	n. sp.	-	n. sp.	✗	n. sp.	✗	n. sp.	✗	**Peer teachers**	**POCUS** • Performance of at least 50 examinations in an US laboratory • Independent Practitioner Certification Canada	Theoretical and practical assessment	n. sp.	Theoretical test, Evaluation survey, Self-assessment	-	Learners at all levels preferred peer-to-peer teaching over instruction from staff.

*Abbreviations:* *n* Number, *y°* year of medical school, *pc* preclinical, *c* clinical, *h* hour, *w* week, *m* months, *PT* peer teacher, *FI* faculty instructor, *US* ultrasound, *PoCUS* point of care ultrasound, *TTE* transthoracic echocardiogram, *FoCUS* focus cardiac ultrasound, *FAST* focused assessment with sonography for trauma, *FEEL* Focused Echocardiography in Emergency Life Support, *UME* undergraduate medical education, *P* patients, *HM* healthy models, *n. sp.* not specified

### Quality assessment of included studies

The evaluation of the Newcastle–Ottawa Scale (NOS) scores revealed fair study quality in nine of the 20 studies (45%), corresponding to a NOS score of five or six, and poor study quality in eleven studies (55%) with a NOS score of four or less (see Table 4). The analysis of categorial NOS subscores revealed the following: In the selection category, the included studies averagely met three of four criteria. Regarding comparability, the average NOS score was 0.5 out of two possible points. Finally, in the subanalysis regarding the study outcome, the studies achieved an average score of one out of three possible points (see Table 4). Detailed information is provided in Table 5.

**Table 4:** Quality assessment of included studies according to Newcastle-Ottawa Scale for cohort studies modified to didactic teaching studies

**Author**	**Selection** **(max.** 4 criteria**)**	**Comparability** **(max. **2 criteria**)**	**Outcome ** **(max.** 3 criteria**)**	**Fulfilled criteria**
Ahn et al. [26]	4	-	-	4
Ben-Sasson et al. [16]	4	2	1	6
Boivin et al. [6]	2	-	1	3
Celebi et al. [27]	3	2	1	6
Celebi et al. [3]	3	-	1	4
Celebi et al.[28]	4	1	1	6
Dickerson et al. [29]	1	-	-	1
Eimer et al. [19]	1	-	1	3
Hari et al. [15]	4	-	1	5
Knobe et al. [30]	4	-	1	5
Kühl et al. [31]	3	2	1	6
Li et al. [32]	3	-	1	4
Miller et al. [5]	2	-	-	2
Nourkami-Tutdibi et al. [7]	3	-	2	5
Nourkami-Tutdibi et al. [20]	2	-	1	3
Rong et al. [33]	3	-	1	4
Weimer et al. [17]	3	-	1	4
Weimer et al. [2]	3	2	1	6
Weimer et al. [34]	3	1	1	5
Yan et al. [35]	2	-	1	3
**Category score**	2.9	0.5	0.9	

**Table 5:** Newcastle-Ottawa Scale item evaluation of the included studies (*n*=20)

**Selection**		
** 1) Representativeness of the exposed cohort (medical students taught by peer teachers)**		
a) truly representative of the average student in the community		20/20 (100 %)
b) somewhat representative of the average student in the community		0/20 (0 %)
c) selected group of students		0/20 (0 %)
d) no description of the derivation of the cohort		0/20 (0 %)
** 2) Selection of the non-exposed cohort (medical students taught e.g. by faculty instructors)**		
a) drawn from the same community as the exposed cohort		14/20 (70 %)
b) drawn from a different source		0/20 (0 %)
c) no description of the derivation of the non-exposed cohort		6/20 (0 %)
** 3) Ascertainment of exposure (ensuring adequate content, practical, and didactic competence of peer teachers)**		
a) ultrasound course led by peer teachers who were trained by internships		16/20 (80%)
b) ultrasound course led by peer teachers who were trained by course concepts		
c) ultrasound course led by peer teachers who were trained by a different concept		0/20 (0 %)
d) no description of peer teacher training		4/20 (20 %)
** 4) Demonstration that outcome of interest (ultrasound skills of taught students) was not present at start of study**		
a) yes, no prior ultrasound skills were obtained (e.g. 1^st^ or 2^nd^ year of medical school, survey on prior ultrasound experiences)		8/20 (40 %)
b) no description of the presence or absence of any prior ultrasound skills		12/20 (60 %)
**Comparability**		
** 1) Comparability of cohorts on the basis of the design or analysis**		
a) study controls for year of medical school/age		6/20 (39 %)
b) study controls for sex		
c) randomized allocation of study participants		5/20 (25 %)
d) no control for any factors/confounders		9/20 (45 %)
**Outcome**		
** 1) Assessment of outcome **		
a) objective practical skills evaluation of students taught by peer teachers/faculty instructors (e.g. OSCE)		17/20 (85 %)
b) objective theoretical skills evaluation of students taught by peer teachers/faculty instructors (e.g. MCQ)		
c) subjective skills evaluation of students taught by peer teachers/faculty instructors (e.g. self-assessment)		3/20 (15 %)
d) no description		0/20 (0 %)
** 2) Follow-up for long-term outcomes**		
a) yes		1/20 (5 %)
b) no		19/20 (95 %)
** 3) Adequacy of follow-up of cohorts**		
a) complete follow-up (all subjects accounted for)		0/20 (0 %)
b) incomplete follow-up (subjects lost to follow-up unlikely to introduce bias (small number lost) or description provided of those lost)		0/20 (0 %)
c) incomplete follow-up (subjects lost to follow-up likely to introduce bias or no description provided of those lost)		1/20 (5 %)
d) no statement		19/20 (95 %)

Cohort = medical students; exposed cohort = medical students taught by peer teacher; non-exposed cohort = medical students taught by faculty instructors

### Characteristics and requirements of students for becoming peer teachers

Groups of a median of 13 (range: 3 to 44) medical students were trained as peer teachers. Student peer teachers attend the 2nd to 6th year, with a median attendance in the 4th year of medical school (see Table 6). In only one study [5], peer tutors started from a preclinical level, whereas most studies only trained clinical students to become student teachers. Related data were missing in four studies. The background and specific ultrasound experience of peer teachers were generally underreported. Most of the studies did not address whether the students should have any previous teaching experiences or fulfill any further professional or personal requirements to enter the training program. Given the shortage and the difficulty in recruiting peer teachers, many programs do not require applicants to meet any specific criteria to minimize barriers for potential new tutors. In the course conducted by Celebi et al., all students of the third year onwards could apply freely without any further selection criteria [28]. However, a few studies highlighted the importance of prior training in determining peer-assisted learning success. There are peer teacher programs that expect prior teaching experience and completion of a specific introductory course or ultrasound elective [6, 16]. A common concept also requires students to complete the ultrasound course, which they plan to teach [29].

**Table 6:** Number and year of peer teachers and trained students in the context of the duration of the respective national medical curriculum

**Study**	**Country**	**National duration of medical school**	**Peer teachers**		**Trained student cohort**	
			**Number**	**Year**	**Number**	**Year **
**Ahn et al. **[26]	Canada	4	20	4^th^	310	1^st^-2^nd^
**Ben-Sasson et al. **[16]	Israel	6	4	3^rd^	44	1^st^
**Boivin et al. **[6]	USA	4	n. sp.	3^rd^ -4^th^	228	1^st^-2^nd^
**Celebi et al. **[27]	Germany	6	44	3^rd^ -5^th^	n. sp.	n. sp.
**Celebi et al. **[3]	Germany	6	10-16	3^rd^ - 4^th^	n. sp.	n. sp.
**Celebi et al.** [28]	Germany	6	18	3^rd^ -5^th^	75	5^th^
**Hari et al. **[15]	Switzerland	6	16	4^th^ - 6^th^	n. sp.	3^rd^
**Knobe et al. **[30]	Germany	6	3	4^th^	151	3^rd^- 4^th^
**Miller et al. **[5]	USA	4	19	2^nd^	63	2^nd^-3^rd^
**Nourkami-Tutdibi et al. **[7]	Germany	6	4-6	3^rd^ - 4^th^	40	3^rd^-6^th^
**Nourkami-Tutdibi et al. **[20]	Germany	6	30	n. sp.	75	n. sp.
**Rong et al. **[33]	USA	4	11	4^th^	73	3^rd^
**Weimer et al. **[17]	Germany	6	n. sp.	n. sp.	888	3^rd^
**Weimer et al. **[2]	Germany	6	n. sp.	n. sp.	141	6^th^
**Weimer et al. **[34]	Germany	6	6	4^th^ -5^th^	97	3^th^-5^th^
**Yan et al. **[35]	Canada	4	n. sp.	n. sp.	23	2^nd^-3^rd^

### Curricular integration and didactic methods of peer teacher training programs

**Fig. 2:**
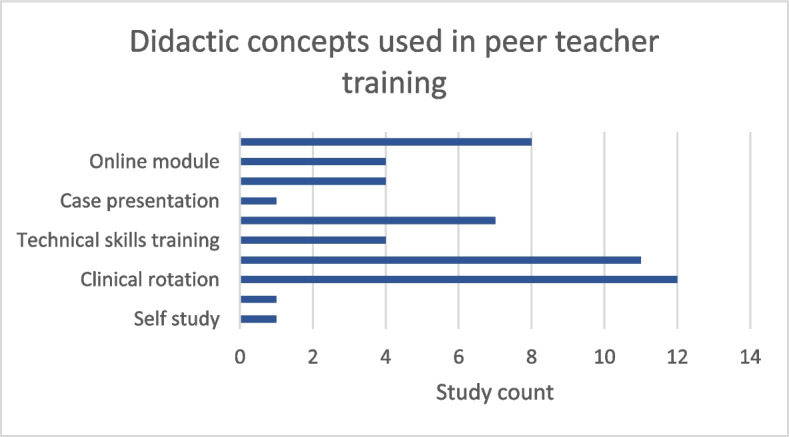
Didactic concepts used in the training of peer teachers

Peer teacher training in ultrasound is implemented in different curricular formats. Internships (50%, 8/16) and courses (94%, 15/16) are prevalent. Didactic concepts used to train medical students as peer teachers included the transfer of knowledge via lectures (57%, 8/14), online modules (29%, 4/14), scripts (29%, 4/14), or case presentations (7%, 1/14). They also involved training of didactic (50%, 7/14) and technical skills (29%, 4/14) during hands-on scanning of healthy volunteers (79%, 11/14), during clinical rotations (86%, 12/14), video reviews of performed scans (7%, 1/14), and self-study of the course content (7%, 1/14) (see Fig. 2). Lectures convey theoretical knowledge, such as ultrasound examination principles and pathophysiological findings. In contrast, didactic training imparts skills and techniques for teaching others, particularly fellow students. This includes training in instructional methods and communication strategies. The median duration of peer teacher training across studies was 10 days (range: 2 to 40). According to a study by Ahn et al. peer teachers who underwent a four-week training were evaluated better than student tutors who were trained for two weeks, indicating an influence of the duration of the training intervention on the teaching quality [26]. When comparing different training approaches, Celebi et al. showed that the training of peer tutors through internships, conventional courses, or a combination of both leads to an equivalent learning outcome for future peer instructors [27]. Subsequently, no significant difference in the effectiveness of their instruction of other students could be measured [28].

### Assessment, certification, and financial remuneration of future peer teachers

In most peer teacher training programs, a final examination evaluates the competence of the future student teachers. Utilized assessment methods included self-assessment and feedback by the course directors (13%, 2/16), as well as objective assessments (56%, 9/16) like multiple choice questionnaires (MCQ) or objective structured clinical examinations (OSCE). In five cases, the form of assessment was either not specified or absent (31%, 5/16). In most studies, students did not receive any certification after completing their training as peer teachers. In the program of Celebi et al., however, it was possible to obtain a certificate from the German Society for Ultrasound in Medicine (DEGUM) after completing the training program [3]. Only five studies addressed whether the students were paid by the university or clinic for their work as peer teachers [3, 5, 6, 26, 33]. Two studies rewarded the students financially with payment or a voucher [3, 6]. The remaining studies relied on the voluntary contribution of the students [5, 26, 33].

### Implementation and evaluation of peer-assisted learning

#### Study design, control, and effect assessment of the educational intervention

Study characteristics of included studies, incorporating educational interventions utilizing peer teaching in undergraduate ultrasound education, were examined following the PICOS scheme (see Table 2). All 20 studies included in this systematic review were found to be prospective in design. The educational interventions involved a median number of 75 (range: 40 to 310) participants. The distribution of participants according to their medical school year revealed most students were in 4th year, but spanning from 1st to 6th year. However, the average difference between years of medical school completed by peer teachers compared to their taught students amounted to 0.95 years. 11% (2/18) of studies included participants in the preclinical study phase, 72% (13/18) involved participants in the clinical phase, and 17% (3/18) included participants in both phases. Two studies did not specify the study year of the participating students. Among examined studies, 70% (14/20) compared their intervention with a control group, whereas in 30% (6/20) no control group was employed.

To measure the effects of didactic approaches involving peer teaching in undergraduate ultrasound education, diverse evaluation methodologies were utilized. In terms of the timing, 75% (15/20) of the studies conducted a post-interventional assessment, whereas 25% (5/20) employed a pre- and post-interventional design. Various modalities were used to assess participants’ knowledge and skills in ultrasound. The theoretical knowledge was assessed in 45% (9/20) of the studies, using multiple-choice questionnaires (MCQ) in five cases. Practical skills assessment was conducted in 70% (14/20) of the studies, using Objective Structured Clinical Examination (OSCE) in twelve cases. The perceived efficacy and acceptability of the implemented ultrasound curriculum, the performance of the peer teachers, the subjective learning success, and further aspects were evaluated by feedback-surveys in 75% (15/20) of the studies.

#### Peer teacher vs. faculty instructors

**Fig. 3:**
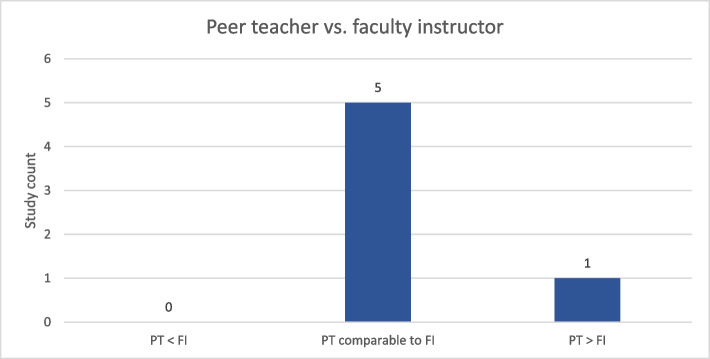
Comparison of performances and outcomes in courses led by peer teachers and faculty instructors Abbr.: PT: peer teacher, FI: faculty instructor

In terms of comparing peer teacher-led courses with faculty instructor-led courses, six studies conducted such comparisons. Among these, the instruction by peer teachers was found to be equivalent to that of faculty instructors in 73% (5/6), while they were deemed superior in 27% (1/6) of the studies (see Fig. 3). Ten studies did not compare directly between peer and faculty-led teaching.

However, when analyzing subitems of ultrasound skills, peer teachers proved not to be equivalent effective with faculty in all aspects of ultrasound training. In a study by Li et al., basic ultrasound principles and physics seemed to be more effective when taught by ultrasound experts than by peer students who focused on maximal hands-on scanning time without beginning with a theoretical introduction [32]. Ahn et al. identified ocular ultrasound as more difficult to be taught by peer teachers [26].

#### Peer teaching in different ultrasound applications and student groups

Ultrasound applications varied, encompassing point-of-care ultrasound (PoCUS), transthoracic echocardiogram (TTE), focused cardiac ultrasound (FoCUS), focused assessment with sonography for trauma (FAST) and abdominal ultrasound (Abdominal US). In three studies, the ultrasound application was not specified [3, 27, 28]. Furthermore, specific protocols such as the Trinity hypotensive ultrasound protocol can be successfully and sustainably taught by peer teachers, as a study by Jeppesen and Bahner showed [36]. The evaluation of student perceptions after the training of different ultrasound applications by peer tutors and faculty instructors identified ocular ultrasound as more difficult to be taught by peer teachers [26]. Ahn et al. concluded that the peer tutors would have needed more intensive training as they only received limited training in ocular ultrasound [26]. Kühl et al. came to a similar conclusion when conducting a peer tutor-based echocardiography course. Students who were instructed by faculty instructors achieved a better average performance than peer-teacher-instructed fellow students [31]. In musculoskeletal ultrasound, the comparison of student and professional teachers showed the superiority of faculty in teaching preclinical students without prior anatomical knowledge [30].

## Discussion

### Characteristics of peer teachers and training approaches

This systematic review aims to provide a comprehensive overview of current studies on peer teaching in undergraduate medical ultrasound education. The analysis of proposed didactic approaches used in the training and assessment of medical students as peer teachers revealed peer teachers averagely have completed approximately one additional year of medical school compared to their taught students. The curricular integration of peer-tutored training showed the predominance of course-based training sessions, followed by internships. Course-based concepts offer to train a considerable number of peer-student tutors simultaneously and allows for a focused delivery of content while maintaining full control over the educational material. On the contrary, the internship model provides a practical focus with ample opportunities for hands-on scanning of patients, enabling students to see first pathologies, while the range of taught content depends on the clinical availability of patients. Unfortunately, the number of students in this approach is constrained by the capacity of the ultrasound laboratories and suitable patients [4, 27]. The study by Celebi et al. demonstrated an equivalence learning outcome for medical students qualified as peer teachers through an internship, course concept, or a combination of both. Thus, the didactic effectiveness of all these modalities appears to be comparable [27].

The median duration of peer teacher training was around ten days, striking a balance between providing substantial education in the constrained setting of the medical curriculum. The positive correlation between the duration of training and peer teachers’ skill acquisition emphasizes the positive effect of allocating more resources to ultrasound education [26]. The final evaluation of peer teachers predominantly leaned towards rather objective assessments than subjective evaluations as they offer a more standardized and impartial measurement of the peer teachers’ competency.

### Comparative effectiveness: benefits and limitations of peer teaching

Most studies comparing peer teaching with faculty-led courses revealed that peer-assisted learning was considered either comparable or even more effective. Peer teachers can significantly improve practical ultrasound training by enabling low student-instructor ratios [6]. Given the high costs of employing ultrasound experts, peer teachers can offer a cost-effective opportunity to ensure practical ultrasound training without having to rely on the voluntary engagement of overburdened clinicians. Furthermore, from a psychological perspective, the higher cognitive and social congruence between peer teachers and students promote a pleasant learning environment. Students may feel more comfortable asking questions and participating actively when instructed by peers [5, 15, 35]. Additionally, peer teachers are less susceptible to the “curse of knowledge”, a cognitive bias hindering effective knowledge transfer from experienced individuals to novice learners. According to this theory, a deeper understanding of certain issues can make it more difficult to explain concepts to new learners in a simple and understandable way, as deeper neurobiological connections can lead to more complex perspectives to the respective topic than would be appropriate for beginners [16, 37]. At this point, peer teachers can bridge this gap and enhance communication. Moreover, studies by Nourkami-Tutdibi et al. and Weimer et al. explored the long-term effects of peer-teaching, leading to sustained improvements in ultrasound skills and knowledge [2, 7]. Nevertheless, peer-assisted learning also has its limitations. According to Li et al. the superiority of peer teachers over faculty experts with the level of prior knowledge of the students being taught depended on level of theoretical knowledge [32]. Nevertheless, both studies still concluded the practicality of peer teaching concepts. While peer-assisted learning offers a valuable approach for many ultrasound applications, it may reach its limits for more complex examinations such as ocular ultrasound or echocardiography [26, 31]. In addition, results of Knobe et al. indicate that peer-teaching concepts should be used primarily in clinical courses with pre-existing anatomical knowledge [30]. This is also supported by a meta-analysis on peer-teaching in medical school by Brierley et al., reporting significant improvement in skills was only achieved through peer-teaching in randomized studies in clinical courses [38]. In addition, professional instructors appear to be more effective for teaching basic principles of ultrasound [38]. Further limitations involve peer teachers’ less experience in teaching and clinical practice resulting in less authority than expert tutors [33].

### Personal benefits for peer tutors

Students who become peer teachers can gain valuable personal benefits [5, 26, 39]. Due to the intensive training, peer tutors gain a deeper understanding of ultrasound than fellow students [5]. In addition, many training programs also include didactic training to strengthen the students’ teaching skills. Although it is not part of the standard medical curriculum, the education of students and residents is increasingly seen as a core competency of every physician [19, 40]. When students volunteer as peer teachers, they acquire teaching skills earlier on that they can benefit from later in their careers. Besides increasing the students´ clinical and didactical competence, peer teaching also has the potential to improve their communication and leadership skills [39]. Furthermore, the formation of student initiatives in ultrasound teaching can also strengthen organizational and political skills and create research opportunities for motivated students in ultrasound teaching [41]. The instruction of other students can strengthen peer tutors in their ability to reflect on their abilities and limitations – a quality that is essential for everyday clinical practice to refer patients to specialists outside their own expertise [42].

### Future directions

Overall, peer teacher-led ultrasound courses offer a promising approach with both benefits and challenges. Nevertheless, it can be stated that the literature still lacks sufficient data to guide the training of ultrasound peer-student tutors. Furthermore, it should be noted that according to our modified version of the Newcastle–Ottawa scale, approximately only half of the included studies can be classified as *fair* while remaining studies presented with poor study quality. Moreover, follow-up investigations to record long-term outcomes are lacking in most studies. Given the limited NOS scores, the number of studies in the field of peer-teacher-led ultrasound teaching is scarce and needs to be expanded. Future studies should demonstrate higher quality, particularly concerning the items of comparability and follow-up studies.

### Limitations

This review has certain limitations. In the course of literature searching, there is an inherent risk of missing relevant articles and incomplete retrieval of pertinent studies, potentially leading to their exclusion from the data synthesis. Given the dynamic nature of ongoing research, additional studies on peer teaching in undergraduate ultrasound education may have been published after the completion of our literature search and before the publication of this paper. In addition, studies specific to osteopathic programs were not included, which could affect the generalizability of findings. Due to the inconsistent study designs and multidisciplinary nature of the studies, the teaching concepts examined can only be compared and translated to each other to a limited extent. Despite these limitations, our systematic review provides a comprehensive overview of the available evidence, offering valuable insights into the role of peer teaching in this educational context.

## Conclusion

This review underscores the prevalent utilization of peer teaching as a solution to resource constraints and educational gaps. Most studies that compared student and postgraduate teachers considered peer teachers equivalent to expert-led courses. This finding emphasizes the efficacy and acceptance of peer teaching in ultrasound education. Peer-assisted learning enhances both peer teachers’ and learners’ academic knowledge, hands-on skills, and interpersonal competencies such as communication and confidence, which are applicable in future clinical situations [5, 19, 35]. Nevertheless, further investigation of training programs to prepare students for the role as ultrasound teachers is needed. Moving forward, concerted effort is required to address gaps in the literature, refine training methodologies, and establish standardized practices, ensuring the continued integration and effectiveness of peer teaching in ultrasound education. Key elements for potential standards could include a structured curriculum that outlines core content areas such as ultrasound physics, anatomy, and practical scanning techniques. Additionally, establishing minimum training hours and providing competency-based assessments to evaluate both theoretical knowledge and practical skills could enhance the quality of peer teacher preparation. Finally, implementing standardized assessment methods, would ensure that peer tutors are equipped with the necessary expertise and confidence to lead ultrasound training. Future research should also explore optimal peer tutor-to-student ratios to balance engagement and effectiveness, the long-term retention of skills taught through peer-led training, and the comparative impact of different teaching modalities. Investigating these areas could help refine peer teaching methodologies and broaden the spectrum of available programs, accommodating different learning preferences and institutional resources. Addressing these aspects will strengthen the integration of peer teaching in ultrasound education, enhancing its sustainability and impact across medical training programs.

## Data Availability

Data available within the article or its supplementary materials.

## Appendix

**Table 7:** Newcastle-Ottawa scale for cohort studies modified to didactic teaching studies

**Selection**	**1) Representativeness of the exposed cohort (medical students taught by peer teachers)**
	a) truly representative of the average student in the community
	b) somewhat representative of the average student in the community
	c) selected group of students
	d) no description of the derivation of the cohort
	**2) Selection of the non-exposed cohort (medical students taught e.g. by faculty instructors)**
	a) drawn from the same community as the exposed cohort
	b) drawn from a different source
	c) no description of the derivation of the non-exposed cohort
	**3) Ascertainment of exposure (ensuring adequate content, practical, and didactic competence of peer teachers)**
	a) ultrasound course led by peer teachers who were trained by internships
	b) ultrasound course led by peer teachers who were trained by course concepts
	c) ultrasound course led by peer teachers who were trained by a different concept
	d) no description of peer teacher training
	**4) Demonstration that outcome of interest (ultrasound skills of taught students) was not present at start of study**
	a) yes, no prior ultrasound skills were obtained (e.g. 1^st^ or 2^nd^ year of medical school, survey on prior ultrasound experiences)
	b) no description of the presence or absence of any prior ultrasound skills
**Comparability**	**1) Comparability of cohorts on the basis of the design or analysis**
	a) study controls for year of medical school/age
	b) study controls for sex
	c) randomized allocation of study participants
	d) no control for any factors/confounders
**Outcome**	**1) Assessment of outcome **
	a) objective practical skills evaluation of students taught by peer teachers/faculty instructors (e.g. OSCE)
	b) objective theoretical skills evaluation of students taught by peer teachers/faculty instructors (e.g. MCQ)
	c) subjective skills evaluation of students taught by peer teachers/faculty instructors (e.g. self-assessment)
	d) no description
	**2) Follow-up for long term outcomes**
	a) yes
	b) no
	**3) Adequacy of follow-up of cohorts**
	a) complete follow-up (all subjects accounted for)
	b) incomplete follow-up (subjects lost to follow-up unlikely to introduce bias (small number lost) or description provided of those lost)
	c) incomplete follow-up (subjects lost to follow-up likely to introduce bias or no description provided of those lost)
	d) no statement

A study can be scored a maximum of one star for each numbered item within the Selection and Exposure categories. A maximum of two stars can be given for Comparability

cohort = medical students; exposed cohort = medical students taught by peer teacher; non-exposed cohort = medical students taught e.g. by faculty instructors

**Table 8:** Scoring algorithm of Newcastle-Ottawa scale for cohort studies modified to didactic teaching studies

**Quality rating**	**Category**			**Total fulfilled criteria**
	**S****election**	**Comparability**	**Outcome**	
Good	≥3	≥2	≥2	≤4
Fair	2	≥1	≥2	5-6
Poor	0-1	0	0-1	≥7
